# Bystander Expression of Atypical Chemokine Receptor 2 Protects T Cells from Chemoattraction towards Cancer‐Associated Fibroblasts

**DOI:** 10.1002/eji.202451215

**Published:** 2025-02-11

**Authors:** Richard Tang, Szun S. Tay, George Sharbeen, David Herrmann, Janet Youkhana, Paul Timpson, Phoebe A. Phillips, Maté Biro

**Affiliations:** ^1^ EMBL Australia, Single Molecule Science node, School of Biomedical Sciences The University of New South Wales Sydney NSW Australia; ^2^ Pancreatic Cancer Translational Research Group, School of Biomedical Sciences, Faculty of Medicine and Health, Lowy Cancer Research Centre The University of New South Wales Sydney NSW Australia; ^3^ Cancer Ecosystems Program The Garvan Institute of Medical Research and The Kinghorn Cancer Centre Darlinghurst NSW Australia; ^4^ School of Clinical Medicine St Vincent's Healthcare Clinical Campus UNSW Medicine & Health, UNSW Sydney Sydney Australia

**Keywords:** ACKR2, cancer‐associated fibroblasts, chemokine scavenging, CXCL1, CXCL12

## Abstract

Atypical chemokine receptors (ACKRs) are a subclass of chemokine receptors that internalise and degrade chemokines instead of eliciting chemotaxis. Scavenging by ACKRs reduces the local bioavailability of chemokines and can thus reshape chemokine gradients that direct leukocyte trafficking during inflammation and anticancer responses. In pancreatic ductal adenocarcinoma (PDAC), chemokine axes, such as CXCL12‐CXCR4, are co‐opted by cancer‐associated fibroblasts (CAFs) for tumour growth and escape, and immunosuppression. Here, we explore the use of ACKRs to reshape chemokine gradients within the PDAC tumour microenvironment. ACKR2, previously only known to scavenge inflammatory CC chemokines, was recently shown to be able to interact with CXCL10 and CXCL14. Here, using a chemokine binding assay and cytometric bead arrays, we reveal that ACKR2 scavenges additional CXC chemokines CXCL12 and CXCL1. ACKR2 scavenges CXCL12 with reduced efficiency compared to ACKR3, previously reported to bind CXCL12. Finally, we demonstrate that the overexpression of ACKR2 on bystander cells protects primary murine cytotoxic T lymphocytes from PDAC CAF‐mediated chemoattraction. These findings reveal new CXC chemokine ligands of ACKR2 and indicate that ACKR overexpression may protect T cells from misdirection by CAFs.

## Graphical abstract

Cancer‐associated fibroblasts (CAFs) secrete chemokines that attract T cells. Here we show that the overexpression of atypical chemokine receptor 2 (ACKR2) by bystander cells scavenges key chemokines secreted by CAFs, including previously unreported ligands CXCL12 and CXCL1, and thereby protects T cells from chemoattraction by CAFs.

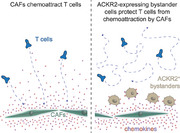

**Abbreviations**
ACKR: atypical chemokine receptor
CAF: cancer‐associated fibroblast
CTL: cytotoxic T lymphocyte
HEK: human embryonic kidney cell 293T
PDAC: pancreatic ductal adenocarcinoma

## 1 Introduction

Chemokines are a large family of small chemotactic cytokines that direct leukocyte migration [1]. Chemokines are divided into subfamilies, namely CC, CXC, XC, and CX3C chemokines, and they have complex, promiscuous, and redundant binding interactions with their corresponding G protein‐coupled chemokine receptors [2]. Neither the expression nor the surface localisation of chemokine receptors is fixed; changes in the expression of specific chemokine receptors confer fate and functional specificity to immune cells [3], and the distribution of chemokine receptors transiently rearranges on the surface of cells in the presence of ligand gradients [4]. In addition to “functional” chemokine receptors, there exists a distinct subclass of chemokine receptors, termed atypical chemokine receptors (ACKRs), that do not chiefly signal to induce chemotaxis; instead, they internalise and degrade chemokines [5]. Atypical chemokine receptor 2 (ACKR2), formerly known as D6 or CCBP2, is a promiscuous receptor for a wide variety of inflammatory CC family chemokines and plays a key role in the resolution of inflammation [6]. Atypical chemokine receptor 3 (ACKR3), also termed CXCR7 or RDC1, is a more specific atypical receptor with the only known ligands being CXCL11 and CXCL12 [7]. Chemokines have been heavily implicated in cancer progression, although their role is controversial as they induce both pro‐ and anti‐tumour responses [8]. The chemokine axis CCR5‐CCL3/CCL4/CCL5 has been shown to promote cytotoxic T lymphocyte (CTL) mass recruitment and infiltration of solid tumour masses [9]. High CTL infiltration of pancreatic ductal adenocarcinoma (PDAC) tumours is associated with better patient outcomes [10]. By contrast, other chemokine axes, such as the CXCL12‐CXCR4/ACKR3 pathway, are protumourigenic as they promote cancer proliferation, angiogenesis, metastasis and/or chemotherapy resistance [7]. Whereas ACKR3 is not detectable in healthy pancreatic tissue, in the majority of PDAC patients, ACKR3 is expressed by ductal cells, but not in acinar or stromal tissue [11]. Importantly, the CXCL12‐CXCR4 axis has been shown to sequester CTLs in the PDAC stroma, preventing tumour infiltration and clearing by CTLs [12].

PDAC has a unique stroma that causes CTLs to be trapped at the tumour periphery, resulting in immune exclusion [13]. Historically thought to act as a simple physical barrier, the fibrotic PDAC stroma has been recharacterised to include complex chemokine interactions between reprogrammed stromal cells, such as cancer‐associated fibroblasts (CAFs), and immune cells, to establish a microenvironment favourable for tumour progression [14]. CAFs are dysregulated fibroblasts that not only secrete and contract extracellular matrix to remodel the PDAC stroma, but also co‐opt chemokine signalling to facilitate tumour growth, metastasis, and recruitment of immunosuppressive immune cells [15].

Consequently, recent approaches in PDAC treatment have utilised a combinatorial approach with immunotherapies and chemokine inhibitors that target protumourigenic chemokine axes upregulated in CAFs [16]. For instance, a clinical trial for BL‐8040, a CXCR4 inhibitor, in combination with immune checkpoint inhibition, showed improved CTL tumour infiltration, decreased immunosuppressive immune cells, and improved overall PDAC patient survival [17]. Presently, chemokine inhibitors only exist for a limited number of signalling axes, and beneficial responses are often short‐lived, which contrasts with the durable responses seen in immunotherapies targeting haematopoietic malignancies [10]. The use of ACKRs remains a poorly characterised and understudied approach that may offer cell‐based alternative to small‐molecule inhibitors, providing a novel approach to reshape chemokine gradients within the PDAC tumour microenvironment to restore the navigational freedom of T cells.

## 2 Results and Discussion

### 2.1 ACKR2 Binds and Internalises CCL3 and CXCL12

**FIGURE 1:**
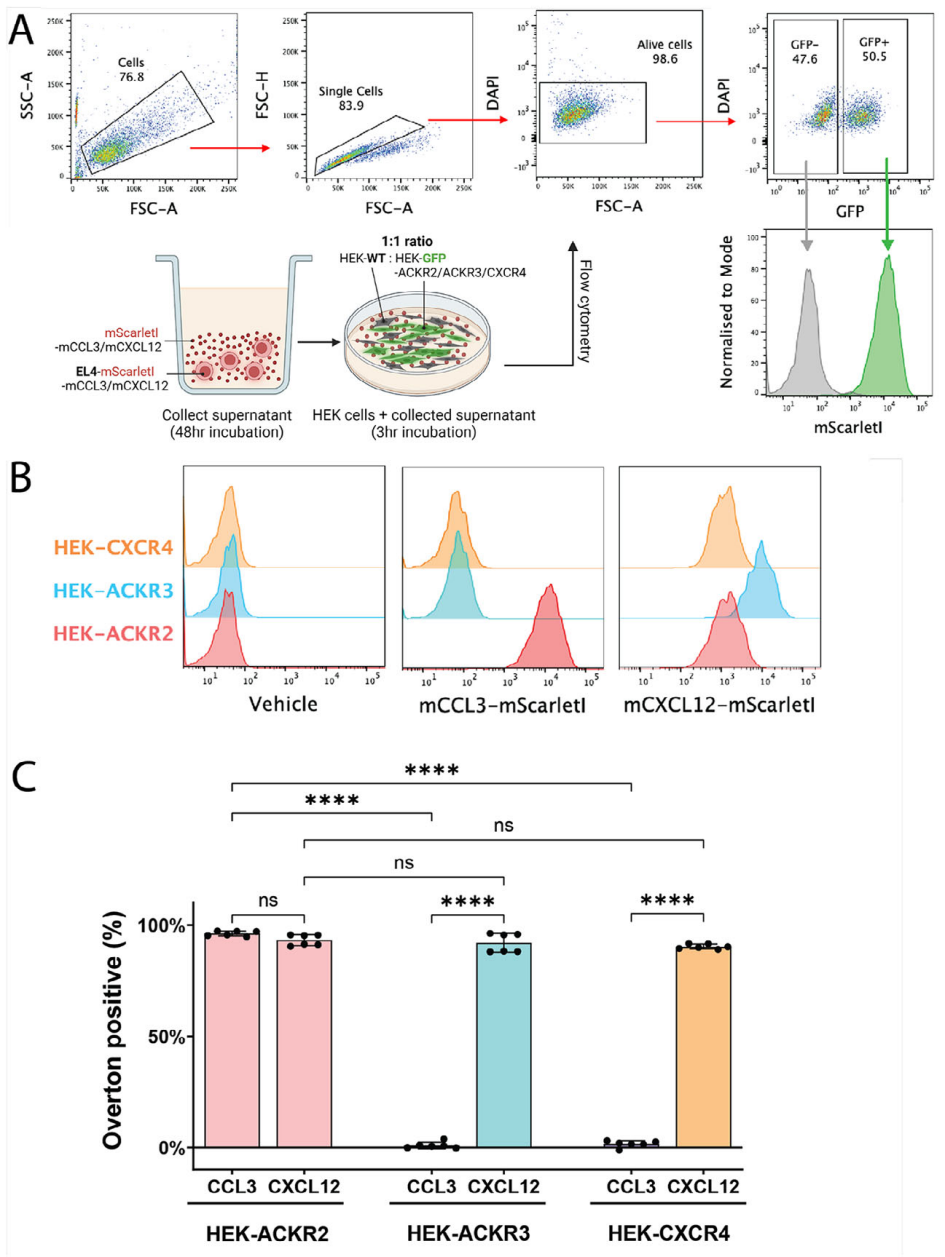
mScarletI fusion chemokines bind to ACKR2, ACKR3, and CXCR4‐expressing HEK cells. (A) mScarletI fusion chemokines were obtained from EL4‐mScarletI‐mCCL3/mCXCL12 supernatant after 48 h incubation in culture and concentrated. ∼400 ng of mScarletI chemokines or media were incubated for 3 h with ACKR2, ACKR3, or CXCR4 expressing HEK cells mixed in a 1:1 ratio with HEK‐WT cells to a total of 1 × 10^5^ cells. Cells were washed after incubation and mScarletI fluorescence was quantified via flow cytometry. A representative flow gating strategy is shown. (B) Representative mScarletI fluorescence modal histograms for each receptor and chemokine condition are shown. The Overton cumulative histogram subtraction algorithm (33) was used to determine the percent difference between sample histograms as a quantitative measure of mScarletI chemokine binding to HEK cell receptors. (C) The compiled Overton positive (%) percentage for *n* = 6 samples from two independent experiments is shown with mean ± SD (error bars). *p*‐values by ordinary one‐way ANOVA with Tukey's multiple comparisons test, ns: not significant, **** *p* ≤ 0.0001.

We studied decoy chemokine receptors ACKR2 and ACKR3 for their roles as a promiscuous scavenger of CC chemokines and a more specific scavenger of CXCL12, respectively, with CXCR4 as a specific cognate control receptor for CXCL12 [2] (see Supporting Information 1 for Materials and Methods). We designed fusion constructs with EGFP linked to murine chemokine receptor sequences, which were packaged into retrovirus and used to transduce HEK293T (HEK) cells (Supporting Information 2). The small monomeric fluorescent protein mScarletI was similarly fused to murine chemokines CXCL12 and CCL3 (mCXCL12‐mScarletI, mCCL3‐mScarletI), and transduced into EL4 cells as previously described [9] (Supporting Information 3). CCL3 and CXCL12 were selected as exclusive, non‐promiscuous ligands for ACKR2 and ACKR3, respectively. We then examined the surface expression and chemokine binding specificity of transduced HEK cells by incubating with mCCL3‐mScarletI or mCXCL12‐mScarletI supernatant collected from transduced EL4 cells (Figure 1A).

Un‐transduced HEK cells (HEK‐WT), serving as an internal non‐binding control population, were mixed in a 1:1 ratio with EGFP‐receptor fusion‐expressing HEK cells prior to the addition of mScarletI‐tagged chemokines. The addition of mScarletI chemokines did not increase mScarletI fluorescence intensities of the GFP‐negative HEK‐WT population throughout, showing that non‐specific binding was not observed in this assay (Supporting Information 4). As expected, samples recapitulating previously known receptor‐ligand interactions (CCL3‐ACKR2 and CXCL12‐ACKR3/CXCR4), exhibited a marked increase in mScarletI fluorescence compared to chemokine‐free media (Figure 1B). Additionally, mCXCL12‐mScarletI fluorescence was increased for ACKR3 compared to CXCR4, corresponding to a higher binding efficiency that corroborates ACKR3's known higher affinity for CXCL12 [18]. Notably, ACKR2 was shown to bind mCXCL12‐mScarletI for the first time, with no significant differences in Overton positive (%) when compared with known receptors, ACKR3 and CXCR4 (Figure 1C).

The discovery that ACKR2 binds CXCL12 was unexpected, as ACKR2 has historically been classified as a receptor for only proinflammatory CC chemokines. However, a recent study revealed via the detection of downstream β‐arrestin‐1 signalling that CXCL10, previously only known to bind to CXCR3, is also a strong agonist of ACKR2 [19]. Additionally, CXCL2 and CXCL12 also appeared to trigger β‐arrestin‐1 recruitment but did not reach statistical significance [19]. Interestingly, another study found that ACKR2 was able to mediate CXCL14‐derived fibroblast responses without any significant binding or β‐arrestin signalling observed [20]. These recent findings highlight our incomplete mapping of ACKR2 ligands, as well as possible unconventional chemokine receptor‐ligand interactions.

### 2.2 ACKR2 Scavenges Chemokines Present in CAF Supernatant

**FIGURE 2:**
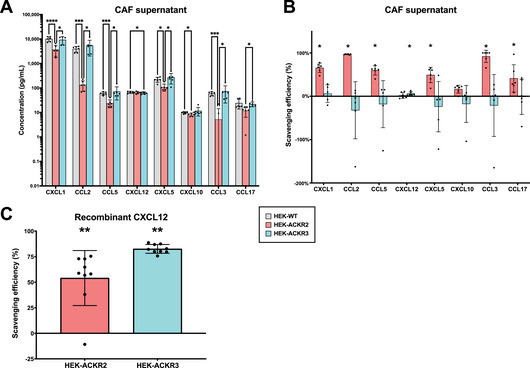
Chemokine scavenging of CAF supernatant and recombinant CXCL12 by ACKR2‐ and ACKR3‐expressing HEK cells. (A) 1 mL supernatant collected from CAFs after 48 h was incubated with 10^6^ ACKR2‐ or ACKR3‐expressing HEK cells, or un‐transduced HEK (HEK‐WT) cells, for 18 h. Chemokine concentrations were subsequently analysed by CBA. Chemokines that were below detectable confidence limits of the CBA assay are not shown. Data is shown as mean ± SD (error bars). *n* = 6 from three independent experiments. *p*‐values by two‐way ANOVA with Tukey's multiple comparisons test, ns not shown, * *p* > 0.05, ** *p* < 0.05, *** *p* < 0.01, ****p < 0.001. (B) Scavenging efficiency (%) was calculated for conditions in (A) by comparing the difference in chemokine concentration of receptor‐expressing HEK cell samples to HEK‐WT concentrations within each experimental run. *p*‐values by Wilcoxon matched pairs two‐tailed test, compared with a theoretical median of 0. ns not shown, *p > 0.05. (C) 10 ng recombinant mouse CXCL12 (rCXCL12) was similarly incubated with HEK cells as above for 12 h. Scavenging efficiency (%) is shown as mean ± SD (error bars). *n* = 9 from three independent experiments. *p*‐values by Wilcoxon matched pairs two‐tailed test, compared to a theoretical median of 0. ns not shown, **p < 0.05.

We next sought to further investigate whether ACKR2 can functionally bind CXCL12 and lead to chemokine degradation (scavenging) in a setting relevant to tumour control. To this end, we isolated CAFs from KPC mice that spontaneously developed PDAC [21, 22] (see Methods in Supporting Information 1), then collected supernatant from the CAFs, which was incubated with HEK‐ACKR2, HEK‐ACKR3, or HEK‐WT cells. Chemokine concentrations in supernatants were measured using cytometric bead arrays (CBA). Of the 14 chemokines profiled in KPC mouse CAF supernatant, CXCL1, CCL2, CCL5, CXCL12, CXCL5, CXCL10, CCL3, and CCL17 were readily detected via CBA (Figure 2A).

Reductions in chemokine concentrations compared with the HEK‐WT incubation control are reported as scavenging efficiencies (Figure 2B, Supporting Information 1). We found that the ACKR2 receptor scavenges known ligands CCL2, CCL5, CCL3, and CCL17, as well as CXCL1 and CXCL5, previously unreported binding partners of ACKR2. CAFs are known to upregulate CXCL1 secretion in the PDAC stroma [23].

As predicted, ACKR3‐expressing HEK cells scavenged CXCL12, albeit at a low efficiency. In this experiment, ACKR2 was not found to significantly scavenge CXCL12. We hypothesised that the low CXCL12 scavenging efficiency of ACKR3 and lack of scavenging by ACKR2 was due to the relatively low concentrations of CXCL12 in the CAF supernatants, which has previously been shown to impede the scavenging of certain chemokines in mouse lung model [24]. ACKR2 is also known to be recycled to the plasma membrane at a higher rate when higher chemokine concentrations are present in a dose‐dependent manner [25]. To address this, we incubated HEK cells with recombinant murine CXCL12 at 10 ng/mL, a concentration that greatly exceeds that detected in the CAF supernatant. Significant scavenging of recombinant CXCL12 by both ACKR2 and ACKR3 was observed (Figure 2C].

Due to the known heterogeneity of CAFs in cancer, we repeated the above experiment with CAFs isolated from KPC mice using an alternative, previously validated method [26, 27] (see Supporting Information 1), which results in comparable chemokine secretion profiles but with more abundant CXCL12 (Supporting Information 5). We confirmed that ACKR2 scavenges CXCL1, CCL2, CCL5, and that ACKR3 scavenges CXCL12. Interestingly, ACKR2 did not exhibit CXCL12 scavenging in this alternative supernatant either. In the presence of a multitude of ACKR2 ligands, CXCL12 may not be scavenged due to differences in binding affinities, and interference or saturation by more dominant interaction partners of ACKR2.

### 2.3 ACKR2 Reduces CTL Migration towards CAF‐Secreted Chemokine Gradients

**FIGURE 3:**
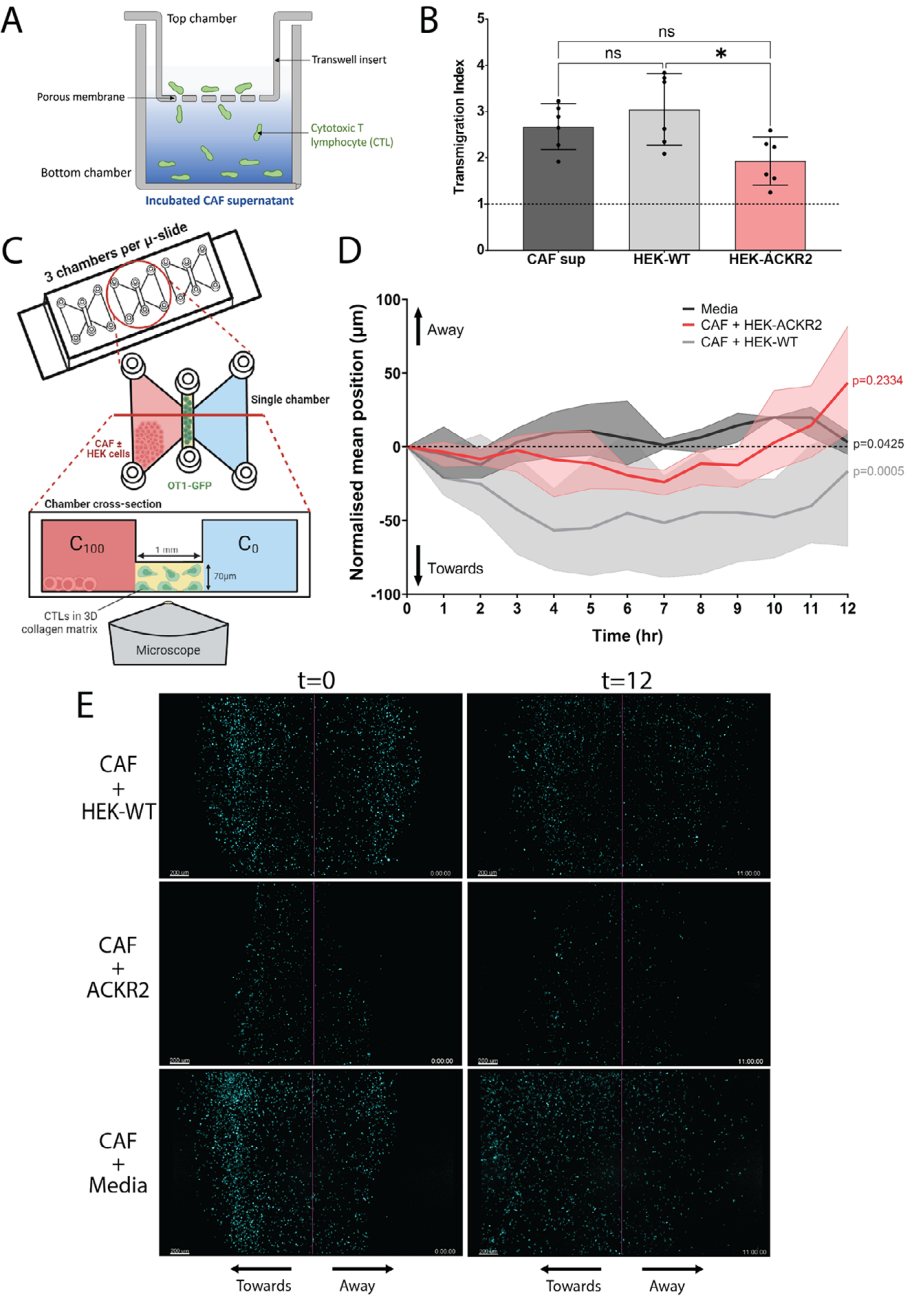
Reduction of CTL chemotaxis towards CAF‐secreted chemokines in the presence of ACKR2‐expressing bystander cells. (A) Schematic showing the principle of the Transwell migration assay. OT‐I GFP T cells [31] (CTLs) are seeded in the top chamber and allowed to migrate through Transwell inserts with 5 µm pores for 3 h. Bottom chambers contained previously collected CAF supernatant (Figure 2A). CTLs added directly to the bottom chamber were used as a control metric for 100% transmigration, and media control was used for baseline transmigration. Transmigrated CTLs were enumerated via flow cytometry. (B) The Transmigration Index was calculated by dividing CTL transmigration by the average transmigration in response to media. Data is shown as mean ± SD (error bars). *n* = 6 from two independent experiments. *p*‐values by ordinary one‐way ANOVA with Tukey's multiple comparisons test. ns p > 0.05, *p < 0.05. (C) OT‐I GFP T cells were embedded in a low‐density collagen matrix in the central chamber of µ‐slide chemotaxis coverslip with CAFs mixed in a 1:1 ratio with either untransduced HEK cells (CAF + HEK‐WT) or ACKR2 expressing HEK cells (CAF + HEK‐ACKR2) seeded in an inlet port. Samples were imaged at 1 h intervals over 12 h. (D) The midline of the central chamber was determined to calculate the mean displacement in the *x*‐axis of all cells. Normalised mean positions (µm) along the *x*‐axis are shown as either towards or away from the inlet port containing the CAF and HEK cells, with negative displacements taken to be towards the inlet port. Data from three independent experiments shown as mean (line) ± SD (shaded area). *p*‐values by two‐way ANOVA with Tukey's multiple comparisons test, comparing means of each condition across time. (E) A representative sample from each condition at 0 h (*t* = 0, left) and 12 h (*t* = 12, right) is shown with CTL fluorescence in cyan and the midlines of chambers shown as solid magenta lines. Scale bars = 200 µm.

To determine if chemokine scavenging can lead to reduced T cell chemotaxis, a Boyden chamber assay was conducted, with CTL transmigration quantified across a 5 µm porous membrane (Transwell insert) in response to scavenged CAF supernatant in the bottom chamber (Figure 3A). A significant reduction in the transmigration index of T cells was found when comparing CAF supernatant incubated with HEK‐ACKR2 and HEK‐WT cells, revealing that chemokine scavenging by ACKR2‐overexpressing HEK cells was sufficient to reduce the chemoattraction of T cells towards CAF secretions (Figure 3B).

We then wanted to investigate the effects of chemokine scavenging by ACKR2 on 3‐dimensional (3D) CTL migration via live imaging within a more physiological environment. To this end, we imaged primary murine CTLs embedded within a 3D collagen matrix in the central channel of a chemotactic chamber where lateral gradients can be established across the channel over a 12 h period (Figure 3C, Supporting Information 6). CAFs together with either HEK‐ACKR2 or HEK‐WT cells were seeded in an inlet port, serving as a chemotactic gradient source in the presence or absence of bystander cells capable of scavenging, respectively. The mean position of CTLs with respect to the midline of the central channel (origin), in response to CAF and HEK cells, or to media, was quantified and normalised to the CTL population's mean position at the onset of the experiment, yielding the normalised mean positions shown in Figure 3D. Figure 3E shows the position of CTLs at the beginning (t0) and conclusion (t12) of the experiment.

At all timepoints, CTLs adjacent to CAFs and non‐scavenging HEK‐WT cells exhibited the greatest bias towards CAF cells, illustrating the potent chemoattraction exerted by CAFs on T cells (Figure 3D). A condition where the inlet ports were loaded with chemokine‐free media served as negative control, and the mean position of CTLs in this condition did not deviate from the channel midline. The mean position of CTLs adjacent to CAFs and HEK‐ACKR2 cells did not significantly differ from that of the media control. These results indicate that ACKR2‐overexpression by bystander cells can redirect CTLs in the presence of CAFs, protecting the T cells from the potent chemoattraction exerted by the CAFs.

## 3 Data Limitations and Perspectives

This study primarily relies on in vitro experiments with ACKRs expressed on HEK cells and isolated mouse CTLs. While this experimental approach allowed us to characterise and gain insight into functional interactions of ACKRs, chemokines, and CTLs, this model does not address the complexities of in vivo host interactions. In particular, the redirection of CTL migration via ACKR2 scavenging was only explored in an isolated in vitro model via confocal microscopy. To address these limitations, further studies could employ a mouse PDAC/CAF model with expression of ACKR2 on other cell types, and evaluation of T‐cell motion via intravital imaging and tracking. Moreover, an interesting extension of this work would involve overexpression of ACKRs on CTLs themselves to create tumour‐homing scavenger cells that are able to specifically absorb misdirecting chemokines present in the tumour microenvironment. Additionally, due to the promiscuity of ACKR2, such a strategy may have even broader applications in autoimmune disorders.

Whilst it is not possible to directly compare the expression levels of ACKR2 we induced in HEK cells in this study to those found in primary human T cells, the differences between control cells (untransduced or unstained) and ACKR2‐expressing cells in our study are comparable to those recently reported during a comprehensive mapping of ACKR expression in various T‐cell subsets from healthy human donors [28].

We were unable to determine the underlying reasons why CAF‐secreted CXCL12, as part of supernatants that include other chemokines, was not scavenged efficiently by ACKR2 (and to some degree ACKR3), despite binding being detected in the binding assay and the ACKRs readily scavenging recombinant mouse CXCL12. It is possible that the CAFs are secreting additional factors (not detected here) that could compete for CXCL12 scavenging. We also show preliminary findings for other novel ACKR2 scavenging interactions, namely with CXCL1 and CXCL5, the functional significances of which require further exploration.

## 4 Concluding Remarks

In summary, we discover that ACKR2 binds and scavenges CXCL12 and CXCL1 and that ACKR2 overexpression in bystander cells can protect CTLs from CAF‐mediated chemoattraction. CXCL12 has been shown to be abundantly secreted by CAFs to confine CTLs to the stroma and suppress their motion into pancreatic tumours. Inhibition of the CXCL12‐CXCR4 axis has resulted in increased T‐cell infiltration of PDAC tumours, and combined with checkpoint blockade, enhances pancreatic cancer therapeutic efficacy [17, 29, 30]. Consequently, our findings lend support to the idea that the overexpression of ACKRs may similarly alleviate the chemotactic sequestration of T cells by CAFs around solid tumours.

## Author Contributions

Richard Tang and Szun S. Tay performed experiments. Richard Tang, Szun S. Tay, and Maté Biro performed data analysis and interpretation. George Sharbeen, David Herrmann, Janet Youkhana, Paul Timpson and Phoebe A. Phillips isolated cancer‐associated fibroblasts and provided feedback on the manuscript. Richard Tang and Maté Biro wrote the manuscript. Szun S. Tay and Maté Biro conceived and supervised the study.

## Ethics Statement

Primary T cells and CAFs were isolated from mice in accordance with the University of New South Wales Animal Care and Ethics Committee (ACEC) protocol 19/133B and Garvan/St. Vincent's Animal Ethics Committee protocol ARA 19/10, respectively, and the Australian Code of Practice for Care and Use of Animals for Scientific Purposes.

## Conflicts of Interest

The authors declare no conflicts of interest.

## Supporting information



Supporting information

## Data Availability

The data that supports the findings of this study are available in the main text and figures and the supplementary material of this article.
