# Supplementary material for: Bystander Expression of Atypical Chemokine Receptor 2 Protects T Cells from Chemoattraction towards Cancer‐Associated Fibroblasts
Source: Eur J Immunol. 2025 Feb 11;55(2):e202451215. doi: 10.1002/eji.202451215 (PMC11811810; doi:10.1002/eji.202451215)
Supplement: Supplementary file 1 — Supporting information [file EJI-55-e202451215-s001.pdf]

Supporting Information

For

Bystander expression of atypical chemokine receptor 2 protects T cells from chemoattraction towards cancer-associated fibroblasts

Richard Tang[1], Szun S. Tay[1], George Sharbeen[2], David Herrmann[3,4], Janet Youkhana[2], Paul Timpson[3,4], Phoebe A. Phillips[2], Maté Biro[1,*]

[1] EMBL Australia, Single Molecule Science node, School of Biomedical Sciences, The University of New South Wales, Sydney, NSW 2052, Australia

[2] Pancreatic Cancer Translational Research Group, School of Biomedical Sciences, Faculty of Medicine and Health, Lowy Cancer Research Centre, The University of New South Wales, Sydney, NSW 2052, Australia

[3] Cancer Ecosystems Program, The Garvan Institute of Medical Research and The Kinghorn Cancer Centre, Darlinghurst, NSW 2010, Australia

[4] School of Clinical Medicine, St Vincent's Healthcare Clinical Campus, UNSW Medicine & Health, UNSW Sydney, Sydney, 2010, Australia

*Correspondence: Maté Biro (mate.biro@emblaustralia.org)

Contents:

Supporting Information 1: Materials and Methods p.2

Supporting Information 2: Chemokine receptor sequences and cloning protocol p.5

Supporting Information 3: Production of mScarletI fusion chemokines p.8

Supporting Information 4: Binding assay histograms p.10

Supporting Information 5: Chemokine secretion profile and ACKR scavenging of supernatant from CAFs isolated with alternative method p.12

Supporting Information 6: Details of chemotaxis migration assay p.13

Supporting References p.16

Supporting Information 1: Materials and Methods

Cells, isolations, and culture

Cancer-associated fibroblasts (CAFs) were isolated from KPC mice (21) and validated as previously described (22). Briefly, CAFs were validated by immunocytochemistry for GFAP and α-SMA positivity, and were negative for cytokeratin.

An alternative CAF isolation method was employed to generate data presented in Supporting Information Figure 2 only: this method, previously validated and described (26, 27), yields CAFs that secrete a chemokine profile with more abundant CXCL12.

Animal work to isolate CAFs was performed in compliance with the Garvan/St. Vincent's Animal Ethics Committee (ARA 19/10) and the Australian code of practice for care and use of animals for scientific purposes.

Human embryonic kidney 293T (HEK) (ATCC# 293T) and EL-4 T lymphoblasts (ATCC# TIB-39) were obtained from ATCC. The HEK-derived *gag/pol* 2 (GP2-293) cell line was obtained from Takara Bio (Kusatsu, Shiga prefecture, Japan). Primary murine CTLs were isolated from OT-I x Lifeact-GFP T cell receptor transgenic mice (32), in accordance with University of New South Wales animal care and ethics committee approval 19/133B. T cells were supplemented with 100 ng/mL recombinant murine interleukin 2 (IL-2) (Peprotech, Rocky Hill, NJ, USA) every 2 days until use on day 6 and 7 post-isolation.

CAF, HEK and GP2-293 cells were cultured in Dulbecco's Modified Eagle Medium (DMEM) supplemented with 2mM L-glutamine, 100U/mL Penicillin, 100U/mL Streptomycin and 10% heat-inactivated fetal calf serum (hi-FCS) (Gibco, ThermoFisher Scientific, Waltham, MA, USA). EL-4 and primary murine CTLs were cultured in T cell media (TCM) composed of Roswell Park Memorial Institute (RPMI) media supplemented with 2mM L-glutamine, 100U/mL Penicillin, 100U/mL Streptomycin, 10% hi-FCS, 1mM sodium pyruvate, 50µM B2-mercaptoethanol and 10mM HEPES (Gibco). All cells were incubated at 37°C and 5% CO_2 and passaged immediately prior to confluency at ~1×10^6 cells/mL, 2-3 times per week.

Creating chemokine receptor expressing HEK cell lines

Murine coding sequences for ACKR2, ACKR3 and CXCR4 were obtained from UniProt, synthesised as gBlocks by Integrated DNA technologies, Inc. (Skokie, IL, USA) and cloned as C-terminal EGFP fusion proteins into a murine stem cell virus (MSCV) retroviral expression vector (Supporting Information 1). The sequence-verified retroviral expression plasmids were co-transfected with a pMD2.G plasmid (a gift from Didier Trono, Addgene 12259) encoding the envelope protein of vesicular stomatitis virus (VSV-G) into GP2-293 packaging cell line for retroviral production **(Supporting information 2)**. Retroviral supernatant was filtered through a 0.45µm polyethersulfone (PES) filter (Merck) and 3 mL was added to 1×10^4 HEK cells for transduction over 72 h. Transduced HEK cells

were sorted for the top 20% highest GFP expressing populations using the BD FACSAria™ III Cell Sorter (BD BioSciences).

mScarletI fusion chemokine binding assay

mCCL3-mScarletI and mCXCL12-mScarletI fusion proteins were obtained as described in Supporting Information 2**.** Chemokine receptor expressing HEK cells were seeded in a 1:1 ratio with HEK-WT cells to a total of 1×10^5 cells in a 96F plate (Corning). 200 µL (~400 ng) of mCXCL12-mScarletI, mCCL3-mScarletI or DMEM media was incubated with HEK cells for 3 h in 37° C and 5% CO_2. Wells were washed with 100 µL 1x PBS before HEK cells were detached using 1x Trypsin-EDTA (0.05% trypsin, 0.685 mM EDTA) and collected into 100 µL of FACS wash buffer (0.02% NaN_3, 2% HI-FCS, 2mM EDTA in 1x PBS) containing DAPI stain (0.2 µg/mL) for flow cytometry (BD LSRFortessa™ X-20 Cell analyser). The proportion of mScarletI chemokine binding to transduced chemokine receptor expressing HEK cells (GFP+) and untransduced HEK-WT (GFP-) cells was analysed by FlowJo™ v10.8.1 (BD Life sciences) using the Overton cumulative histogram subtraction algorithm (33).

Chemokine concentration quantification by cytometric bead array (CBA)

The concentration of chemokines in collected supernatants was quantified by cytometric bead array (CBA) using the pre-defined murine 13-plex BioLegend LEGENDplex™ proinflammatory panel (cat. 740007), containing CC chemokines 2, 3, 4, 5, 11, 17, 20, 22 and CXC chemokines 1, 5, 9, 10, 13, and custom 1-plex CXCL12 kit from the hematopoietic stem cell panel (cat. 740677; CXCL12). Three independent biological replicates (n=3) of CAF supernatant were collected after 48 h incubation in T75 flasks and 72 h incubation in T25 flasks respectively and were filtered through 0.45 µm Millex®-HP syringe filters (Merck Millipore, Burlington, MA, USA). 1 mL of collected CAF supernatant or 10ng of recombinant mouse rCXCL12 (SDF1-β) (BioLegend) diluted in DMEM media was incubated with $1x10^6$ HEK-ACKR2, HEK-ACKR3 and HEK-WT cells in a 12W tissue culture treated plate (Corning) for 18 h at 37°C and 5% CO_2. Incubated supernatants were analysed via CBA to determine chemokine concentrations according to manufacturer instructions. Supplied kit protein standards were used to run a standard curve for the proinflammatory kit and a similar standard curve was run with recombinant mouse CXCL12 (SDF-1β) (BioLegend, cat. 589802) diluted to a top standard of 10ng/mL for the custom 1-plex hematopoietic stem cell kit. Standard curves were fitted using nonlinear regression curve fit, sigmoidal, 4PL, X is concentration model in GraphPad Prism version 10.1.1 (GraphPad Software, San Diego, CA, USA) and chemokine concentrations (pg/mL) were interpolated from these curves.

The percentage loss of chemokines after incubation with receptor expressing HEK cells compared to incubation with HEK-WT cells was defined as 'Scavenging efficiency', and was calculated as:

$$Scavenging\ Efficiency\ (\%) = \left(1 - \left(\frac{[chemokine]\ after\ incubation\ with\ receptor\ expressing\ HEK\ cells}{[chemokine]\ after\ incubation\ with\ HEK-WT}\right)\right) \times 100$$

Boyden Chamber (Transwell) migration assay

CTL transmigration to previously collected incubated CAF supernatant or media was quantified using 6.5mm Transwell® polycarbonate membrane inserts with 5µm pore size (Corning, cat. 3421). 5×10^5 OT1-GFP cells in 100µL of TCM was added to the top chamber and 600µL of KPC CAF supernatant incubated with or without HEK-ACKR2 or HEK WT, or media was added to the bottom chamber of the Transwell. As a total input control, 5×10^5 OT1-GFP cells were added directly to the bottom chambers and incubated without a Transwell insert. After a 3 h incubation at 37°C and 5% CO_2, 500µL of cells were mixed with 100µL of AccuCount blank particles containing 1×10^4 particles (Spherotech Inc, Chicago, IL, USA; cat. ACBP-70-10) and taken for acquisition by flow cytometry (BD LSRFortessa™ X-20 Cell analyser). The number of cells and beads were quantified using FlowJo ™ version 10.8.1. The percentage of CTLs that had migrated through the Transwell insert (Transmigration %)) was calculated as follows:

$$Transmigration\ (\%) = \frac{CTLs\ migrated\ to\ bottom\ well}{total\ CTLs\ added\ to\ control\ well\ without\ inserts}$$

The transmigration index (TI) was defined as CTL transmigration in response to experimental samples, normalised to baseline CTL transmigration in response to media.

$$Transmigration\ Index\ (TI) = \frac{Transmigration\ (\%)\ to\ sample}{average\ Transmigration\ (\%)\ to\ media}$$

Confocal microscopy of 3D chemotaxis assay

CTL migration within three-dimensional (3D) collagen matrices was quantified using µ-Slide Chemotaxis coverslips (Ibidi, Fitchburg, WI, USA, cat. 80326). CAF and HEK-ACKR2 or HEK-WT cells were mixed in a 1:1 ratio to a concentration of 3.3×10^6 cells/mL and 15 µL was pipetted into a side reservoir. 5×10^5 OT-I x Lifeact-EGFP cells (32) were seeded within a collagen gel matrix in the central chamber. A media control where no CAF or HEK cells were seeded was used. The central chamber and immediate surrounding area of all three chambers on the coverslip were imaged concurrently every hour for 12 h in a 37°C and 5% CO2 incubation chamber with a 10x objective using the Leica SP8 DLS (Leica, Wetzlar, Germany). See **Supporting Information Figure 3** for details. Image analysis was performed using Imaris software version 9.2.1 (Bitplane, Belfast, UK) to create Spots corresponding to T cell fluorescence. The midline of the imaged central chamber was set as the origin and the displacement in X from the origin for each Spot was quantified to determine a mean displacement in X, with increasing negative displacement indicating bias towards the experimental condition. The mean displacement of Spots at each time point was then normalised to displacements at 0 h, resulting in the normalised mean position (µm) metric.

Supporting Information 2: Chemokine receptor sequences and cloning protocol

Chemokine receptor sequences were obtained as double stranded DNA fragments (gBlock) from Integrated DNA technologies and cloned into a gammaretroviral expression plasmid (1), with receptor sequences tagged to monomeric enhanced green fluorescent protein (eGFP) (2) by two GS residues and a flexible DPPVAT flexible linker to ensure correct protein folding. The internal methionine start codon in eGFP is replaced with serine to prevent internal translation, ensuring receptor expression is directly correlated with eGFP expression. A detailed protocol of the cloning method is described below.

gBlocks and the retroviral vector were restriction digested using 10U/µg DNA of BamHI-HF, NsiI-HF and/or MluI-HF (New England BioLabs, Ipswich, MA, USA), followed by vector dephosphorylation using 20U of recombinant shrimp alkaline phosphatase (New England BioLabs), before 1% agarose gel excision and purification using the Wizard® SV Gel and PCR Clean-up System (Promega, Madison, WI, USA). gBlocks and vectors were ligated using T4 DNA ligase (New England BioLabs) and transformed into OneShot™ TOP10 Chemically Competent E. Coli (Invitrogen, Waltham, MA, USA) prior to selection on LB Agar or broth containing ampicillin (100µg/mL) (Sigma-Aldrich). An Isolate II Plasmid Mini Kit (Meridian Bioscience, Cincinnati, OH, USA) was used to purify plasmid DNA. DNA quantity and quality was assessed using the DS-11 series spectrophotometer/ fluorometer (DeNovix, Wilmington, DE, USA) and Sanger sequenced at the Ramaciotti Centre for Genomics (UNSW Sydney, Australia). The MSCV forward (cccttgaacctcctcgttcgacc) and eGFP reverse (tggtgcagatgaacttcaggg) primers were used for Sanger sequencing.

GP2-293 transfection was performed using 60µL of neutralised polyethylenimine (PEI) MAX® 4000 (1 mg/mL, Polysciences, Warrington, PA, USA) mixed with 6.8µg of retrovirus expression plasmid and 3.2µg of pMD2.G plasmid in 500µL of 0.9% NaCl. The solution was vortexed for 30 seconds and incubated for 30mins at room temperature before being added to 8mL of $7{\times}10^6$ GP2- 293 cells and incubated for 72hrs at 37°C and 5% CO_2 in 10cm TC-treated dishes (Corning).

Table 1 shows sequences of ACKR2, ACKR3 and CXCR4 used.

Table 1. Chemokine receptor coding sequences.

	GenBank accession	Sequence
ACKR3	NM_001271607.1	gcaagtccggagccctgagagatctcagttgctacaaactgctcagcactgaaggagcctgcagcgctcaccgtcaggaaggcaaaccacagcccaggaagccctgaggtgtggttcccaggaagcatctgaaatgggtctcatccatgccttcctagggctggacttttgagtgttcaaagatgaggccacatgcagaggacaccccacaaatcactcttcacctctggcctaaagacatcagggaaggaaccaggcttgattgcagccctgagtgactgggcaggatggggaaaccttttctggtctccttggttgtcagtcctccgtgtggccagcataaacagcaagagatggccaagagacattaactttggactcaaggagcaggtcacttggtcgctctcctcaagaccatggatgtgcacttgtttgactatgcagagcctggcaactactctgacatcaactggccatgtaacagcagcgactgcattgtggtggacactgtgcagtgtcccaccatgcctaacaagaacgtgcttctgtataccctctccttcatctacattttcatcttcgtgatcggcatgattgccaactctgtggtggtctgggtgaatatccaggctaagaccacaggctacgacacgcactgctacatcttgaacctggccattgcagacctgtgggtcgtcatcaccatccccgtctgggtggtcagtctcgtgcagcataaccagtggcccatgggggagctcacatgcaagatcacacacctcatttctccatcaacctctttgggagcatcttcttcctcgcctgcatgagcgtgga

		ccgctatctctccatcacctacttcaccggcacctccagctataagaagaagatggtacgccgt<br>gttgtatgcatcttggtgtggctgctggccttctttgtgtccctgcctgatacctactacctgaagac<br>ggtcacatctgcttccaacaatgagacctactgcaggtccttctaccccgagcacagcatcaag<br>gagtggctgatcggcatggagctggtctctgtcatcttgggctttgctgtccccttcactatcattg<br>cgatcttctacttcctgctcgctagagccatgtcagcatcaggcgaccaggagaagcacagtag<br>ccggaagatcatcttctcctacgtggtggtcttcctggtatgttggctgccgtaccattttgtggttc<br>tgctggacatcttctccatcttacactacatcccgtttacctgtcagctggagaatgtgctctttaca<br>gcgttgcatgtcacccagtgcctgtccttggtgcactgctgtgtcaaccccgtgctctacagcttc<br>atcaaccgcaactacaggtacgagctgatgaaggccttcatcttcaagtactcggccaaaacag<br>gtctcaccaagctcattgatgcctccagagtgtcagagacagagtactctgccctggaacagaa<br>caccaagtgatccatcattctgcagaggtcgggggacacgtgcatgttgcaaatggggcgg<br>ctgggtcctgtggtttcttcaagaaagcaatgtagctttgggtctggttgcttgagtggtatgaaga<br>ggagaaggcatgtgaccatgcttcctgtctctcattctccagccaagatggctgccgcctggat<br>gcccatcctgacagctggcagctggcagctggcagctggcagcaggcagggctgtgctgtg<br>ctgtgctgtgctgcgctgtgctgtgctgtgcagccagagctgcgtatcaaagccagcaccagg<br>acaggctcttatggacatgtgtacagtagaatcttctgtgtttcttcaagtttttacttggtgacttttt<br>gtatttaagttttaagactttattttctcactatggatgtaccttataaatgtatttgaaagctaaatatat<br>tttaaatattgtatgggaggtgtaaggctgttgtattcagaccatgtaggcctcagattagctggac<br>ttgagttttgactaaggatgacattaattgttagctgatttgaaattatatatataaatataaaaatata<br>tataaatttatgccagtctcggctgaaaggttttatttacaatagttttatatctgtgcaatgtttaatg<br>ctggcacagcatatgaaacaacaactgcccagcaatgcagtttgttacatgaaccatattgtaga<br>gttacacttcgggctgcaaacccgtgaacaagggaaacagagcgttctgttgatttgtaagttatt<br>tttttaataaagattttgtttcctaaaa
ACKR2	NM_021609.4	accagggaagagcgacttccccaaccccaggaaaccaggatgtgtgtcctgagactcctgct<br>cactctgcacgtccctgaagaagagacaacagccactgggccctctcagactccggcggatg<br>tcctggggcaaacagtctggctctgcttgaagacagattctcattagagtcggagagaagcact<br>tgtcccacaactctgcaagcgaggagtcagggcaacaaggagagagaattctgagaagaga<br>ggaaaaagcaaagcagcaggagagctggaggaaaggggagcagaggaaccaacaaacc<br>agcaatccgcagctaaccagttaagcccagctctctagctgggggcagagaccagatcctgc<br>aagcatcagagctcgaggacatgcccaccgttgcttccccactgcctctcaccaccgtcggttc<br>cgagaacagcagctccatctacgactacgactacttagatgatatgaccatcttggtttgcagga<br>aggacgaggtcctgtcctttggaagagtctttctgccggtcgtctacagcctgatcttcgtgctgg<br>gcttggctggaaacctcctcctcctggtggtgttgctccactctgcacctcgaagacggacgat<br>ggagctttacctgctgaacctggccgtctccaacctcttgtttgtagtgactatgcccttctgggcc<br>atctctgtggcctggcattgggtttttggtagtttcctgtgcaaggtgataagcactctctactctatt<br>aacttttactgtggtatcttcttcatcacctgcatgagcctggacaaatacctggagattgtccacg<br>ctcagcctctccacagaccgaaggcccagttcaggaacctgcttctcattgtcatggtgtggatc<br>acatccctggccatctctgtcccagaaatggtctttgtgcagatccaccagaccttagatggtgtg<br>tggcactgctatgcggattttggcggacatgcgaccatttggaagctgtacctgcgcttccagct<br>gaaccttctggggtttctcctcccactcttggccatgatcttcttttactcccgcatcggttgcgttct<br>ggtcaggctgaggccgccaggccagggccgggctctgaggatggccgcggccctggtgat<br>agttttcttcatgctgtggttcccatataacctcaccttgtttctgcactcgttgctggacctgcatgt<br>ctttgggaactgtgagatcagccaccgtctggactatacgttgcaggtgacagagagcctggc<br>cttctcccactgctgcttcaccccggtcctctacgccttctgcagtcaccgcttccgccggtacct<br>gaaggcatttctgtctgtgatgttgagatggcaccaggcacctggcaccccttcctctaaccattc<br>tgagagcagcagggttactgcccaggaagacgtggtcagcatgaatgaccttggggagagg<br>cagtctgaggactcccttaacaagggggagatggggaatacttaggcccgagtgatcagcca<br>cggtctgggaacagcactgctctctctgagggacagcgtgactgtgctgctcgccccagtggtt<br>cccaaccaccagcaggccttactcattactgtctcttcctccttctgcttcctggaccccatcctct<br>ctgctgaaccacttcagctcttcactgatctccctccacttccaccccagcgcttctgtggcttcct<br>ggccctcagcagccaatgaggtcactccactcttagccttcagaccttcaagggccatgtgatc<br>attctgtgactttatctccaccgtactccctcctggtctctgggttcagggcacactggcttccact<br>cgtttctgaagcattccaggttcactccagtagggagagttcagctatgccatctctctggacatc<br>ttcttcgccggcctttctccttgggaagagtttggctcattcctttacttcctggagtcatggctcag<br>atctcaccttgttggtgaggaaggaggcactgtgcccaccctgctcaatgccctgtctatacact<br>gatgctcatgtgcttctgcacacacaatattttcactctgtacagggatttctcctgtcgtgctccct<br>gcagactgcctcgagcccacccacgcaggtgagctgcagagtaaggctctcggtgaacagct

		ccctgctctcctcagactcctaccacagaagcttgtgtgtggaaagcctttggaaagctgctgtg<br>gaatgtgagttagcactgtgcatttctggggacagtcacaggttctccatcctccagggcccctttt<br>ctctgtagctcagacaggaggatactttctgcccttgtgtgaggtctagccagtgaatgacccgg<br>acgtgtgcatcccgcaggaggaaggttgggaccatctcagctctggtcttggggactatcagtc<br>aagttcaaagttttagactttacaaaacacatgccagtgagcaggcatccccttaaaacatcctg<br>ctgagaacactaggtatccacatgtagaagattaaagctagattcctgtctctcatctgatacaaa<br>gatcagctaaaattggatcaaggactctaacatatcacctgaagcctcgaaaccatcagagaaa<br>aacacaggggaaacaactaaaaacatcatcagaagcaaggtctttctgagcagagacccccat<br>agccagggaatactcagatcacatgaaattagagtctccgtggtacagagacagggtcagaat<br>gaagagactgtttacagacaacttggccagctttcacctcacggggcccaatattcaaattgtac<br>aggggacaaaaatgttgaataatccaattaaaaaaatgggtaagtgatttgaa
CXCR4	NM_009911.3	aattttgttgcctggtgcagcaggtagcagtgaaacctctgaggcgtttggtgctccggtaacca<br>ccacggctgtagagcgagtgttgccatggaaccgatcagtgtgagtatatacacttctgataact<br>actctgaagaagtgggttctggagactatgactccaacaaggaaccctgcttccgggatgaaa<br>acgtccatttcaataggatcttcctgcccaccatctacttcatcatcttcttgactggcatagtcggc<br>aatggattggtgatcctggtcatgggttaccagaagaagctaaggagcatgacggacaagtac<br>cggctgcacctgtcagtggctgacctcctctttgtcatcacactccccttctgggcagttgatgcc<br>atggctgactggtactttgggaaatttttgtgtaaggctgtccatatcatctacactgtcaacctcta<br>cagcagcgttctcatcctggccttcatcagcctggaccggtacctcgctattgtccacgccacca<br>acagtcagaggccaaggaaactgctggctgaaaaggcagtctatgtgggcgtctggatccca<br>gccctcctcctgactatacctgacttcatctttgccgacgtcagccagggggacatcagtcagg<br>gggatgacaggtacatctgtgaccgcctttaccccgatagcctgtggatggtggtgtttcaattc<br>cagcatataatggtgggtctcgtcctgcccggcatcgtcatcctctcctgttactgcatcatcatct<br>ctaagctgtcacactccaagggccaccagaagcgcaaggccctcaagacgacagtcatcctc<br>atcctagctttctttgcctgctggctgccatattatgtggggatcagcatcgactccttcatcctttg<br>gggtcatcaagcaaggatgtgacttcgagagcatcgtgcacaagtggatctccatcacagag<br>gccctcgccttcttccactgttgcctgaaccccatcctctatgccttcctcggggccaagttcaaa<br>agctctgcccagcatgcactcaactccatgagcagaggctccagcctcaagatcctttccaaag<br>gaaagcggggtggacactcttccgtctccacggagtcagaatcctccagttttcactccagcta<br>acacttatgcaaagacatatataatatatatatatatatatgataaagaactttttatgttacacatt<br>ttccagatataagagactgaccagtcttgtacagttttttttttttattgactgttgggagtttatgttcc<br>tctagtttttgtgaggtttgacttaatttatataaatactttttttgtttgtttgtttcatgtgaatgagtgt<br>ctaggcaggacctgtggccaagttcttagtagctgtttatctgtgtgtaggactgtagaactgtag<br>aggaagaaactgaacattccagaatgtgtggtaaattgaataaagctagccgtgatcctcagct<br>gttgctgcataatctcttcattccgaggagcaccccacccccacccccacccccaccccattctt<br>aaattgtttggttatgctgtgtgatggtttgtttggtttttttttttgttgtttttgtttttgttttttttctgtaaa<br>agatggcacttaaaaccaaagcctgaaatggtggtagaaatgctgggggtttttttgtttgtttgttt<br>tttcagttttcaagagtagattgatttcactccctacaaatgtacagtcttgtattacattgttaataaa<br>agtcaatgataaacttaaaaaaaaa

Supporting Information 3: Production of mScarletI fusion chemokines

mCCL3-mScarletI and mCXCL12-mScarletI fusion proteins were produced via transducing EL-4 as described previously (3). EL4-mCXCL12-mScarletI cells were expanded to 9×10^{8} cells in TCM before being transferred to serum and phenol-free TCM collection media for 48hrs. 90mL of collected supernatant was filtered through 0.45µm Millex®-HP syringe filters (Merck Millipore) and concentrated 750x using Vivaspin® 20, 30,000 MWCO PES filters (Sartorius AG, Göttingen, Germany) for a final volume of 120uL of mCXCL12-mScarletI. 356uL of mCCL3-mScarletI was obtained after being concentrated 421x. The extinction coefficients of mCCL3-mScarletI and CXCL12-mScarletI were calculated from their protein sequence (4, 5) to be 46,215M^{-1} cm^{-1} and 43,110 M^{-1} cm^{-1} respectively, and their absorbance at 280nm with a path length of 1cm was estimated to be 10.19 and 10.41 respectively using the DS-11 series spectrophotometer/ fluorometer (DeNovix, Wilmington, DE, USA). Using the Beer-Lambert law, the concentrations of mCCL3-mScarletI and CXCL12-mScarletI were estimated to be ~16µg/mL and ~45µg/mL respectively.

Table 2 shows sequences of mCXCL12-mScarletI and mCCL3-mScarletI used.

Table 2. mScarletI fusion chemokine coding sequences.

	GenBank accession	Coding sequence
mCXCL12-mScarletI	NM_013655.4	atggacgccaaggtcgtcgccgtgctggccctggtgctggccgcgctctgcatcagtgacggtaaaccagtcagcctgagctaccgatgcccctgccggttcttcgagagccacatcgccagagccaacgtcaagcatctgaaaatcctcaacactccaaactgtgcccttcagattgttgcacggctgaagaacaacaacagacaagtgtgcattgacccgaaattaaagtggattcaagagtacctggagaaagctttaaacaagaggctcaagatgggaggtggaggttccggcggtggtggatccggaggtgggggttccgtgagcaagggcgaggcagtgatcaaggagttcatgcggttcaaggtgcacatggagggctccatgaacggccacgagttcgagatcgagggcgagggcgagggccgcccctacgagggcacccagaccgccaagctgaaggtgaccaagggtggccccctgcccttctcctgggacatcctgtcccctcagttcatgtacggctccagggccttcatcaagcaccccgccgacatccccgactactataagcagtccttccccgagggcttcaagtgggagcgcgtgatgaacttcgaggacggcggcgccgtgaccgtgacccaggacacctccctggaggacggcaccctgatctacaaggtgaagctccgcggcaccaacttccctcctgacggccccgtaatgcagaagaagacaatgggctgggaagcgtccaccgagcggttgtaccccgaggacggcgtgctgaagggcgacattaagatggccctgcgcctgaaggacggcggccgctacctggcggacttcaagaccacctacaaggccaagaagcccgtgcagatgcccggcgcctacaacgtcgaccgcaagttggacatcacctcccacaacgaggactacaccgtggtggaacagtacgaacgctccgagggccgccactccaccggcggcatggacgagctgtacaagtga
mCCL3-mScarletI	NM_011337.2	ctaccctcgtaaaggatccttcgaagatctacgtatgcatacgcgtataccggtgccaccatgaaggtctccaccactgcccttgctgttcttctctgtaccatgacactctgcaaccaagtcttctcagcgccatatggagctgacaccccgactgcctgctgcttctcctacagccggaagattccacgccaattcatcgttgactattttgaaaccagcagcctttgctcccagccaggtgtcattttcctgactaagagaaaccggcagatctgcgctgactccaaagagacctgggtccaagaatacatcactgacctggaactgaatgcctccggaggaggaggatccggaggaggaggatccggaggaggaggatccgtgagcaagggcgaggcagtgatcaaggagttcatgcggttcaaggtgcacatggagggctccatgaacggccacgagttcgagatcgagggcgagggcgagggccgcccctacgagggcacccagaccgccaagctgaaggtgaccaagggtggccccctgcccttctcctgggacatcctgtcccctcagttcatgtacggctccagggccttcatcaagcaccccgccgacatccccgactactataagcagtccttccccgagggcttcaagtgg

		gagcgcgtgatgaacttcgaggacggcggcgccgtgaccgtgacccaggacacctccctggaggacggcaccctgatctacaaggtgaagctccgcggcaccaacttccctcctgacggccccgtaatgcagaagaagacaatgggctgggaagcgtccaccgagcggttgtaccccgaggacggcgtgctgaagggcgacattaagatggccctgcgcctgaaggacggcggccgctacctggcggacttcaagaccacctacaaggccaagaagcccgtgcagatgcccggcgcctacaacgtcgaccgcaagttggacatcacctcccacaacgaggactacaccgtggtggaacagtacgaacgctccgagggccgccactccaccggcggcatggacgagctgtacaagtgaccgcggttaactgcagcgctagcatatgtcgacagtttgtttg

Supporting Information 4: Binding assay histograms

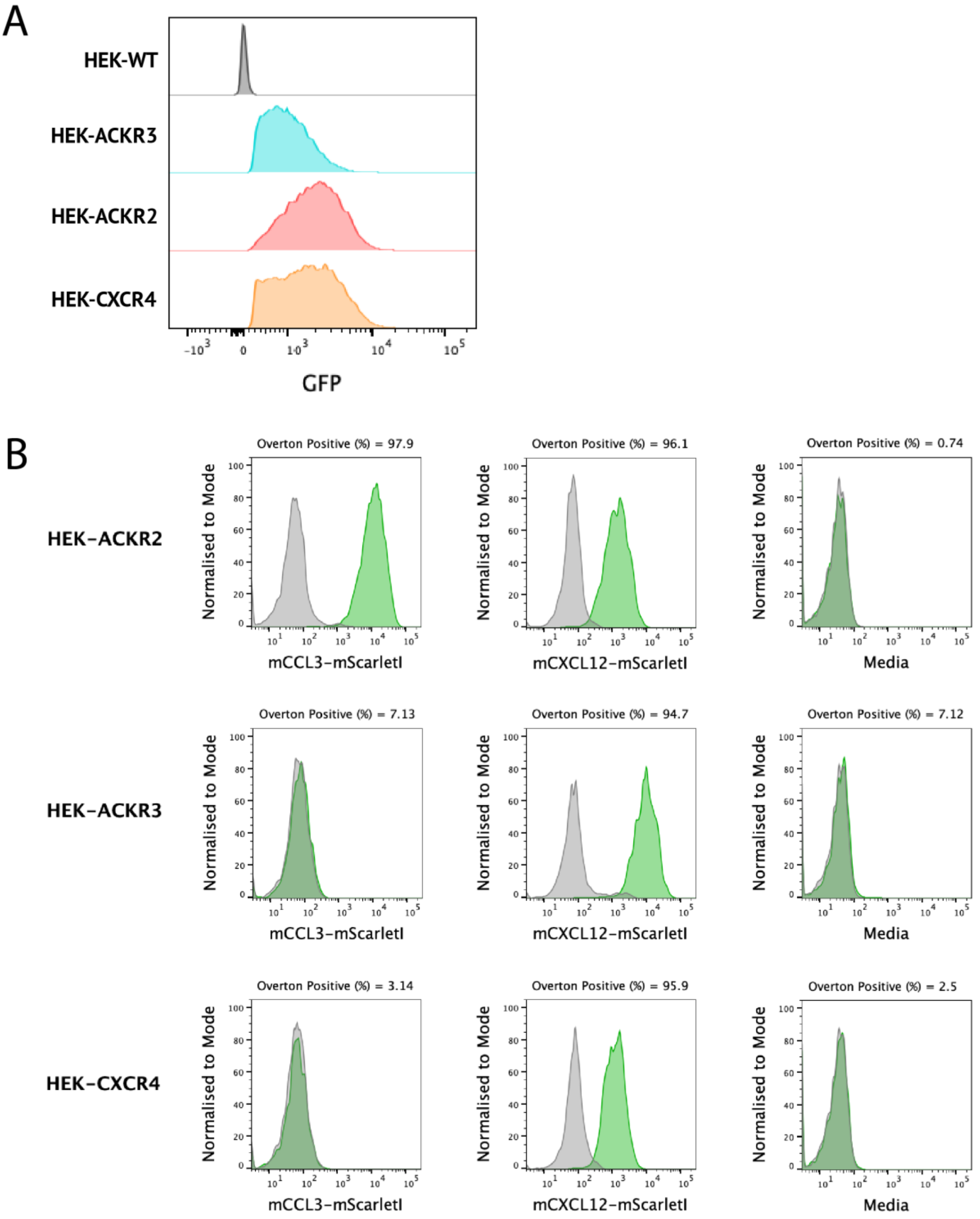

Supporting Information Figure 1. Representative Overton positive histograms for each receptor binding condition. (A) The GFP mean fluorescence intensity (MFI) of transduced HEK cells expressing chemokine receptors compared to HEK-WT is shown. The GFP MFI directly correlates to the amount of receptor expression as the chemokine receptors are linked with eGFP. **(B)** Representative sample showing the flow cytometry gating strategy and Overton cumulative histogram subtraction analysis used to determine HEK-ACKR2 binding with mCCL3-mScarletI or media. HEK cells were

identified by exclusion of dead cells with 4′,6-diamidino-2-phenylindole (DAPI) staining and by GFP fluorescence into receptor expressing (GFP+) and untransduced (GFP-) HEK cell populations. The mScarletI fluorescence of these two populations were then compared using the Overton cumulative histogram subtraction algorithm (6) in FlowJo. (B) Representative overlaid histogram plot showing binding of mCCL3-mScarletI, mCXCL12-mScarletI fusion proteins or media to transduced GFP+ chemokine receptors in HEK cells. mScarletI fluorescence is shown on the x-axis of histograms displaying GFP+ and GFP- populations of HEK cells taken from one representative technical triplicate. The Overton positive (%) metric displays the percent of GFP+ population events that are mScarletI fluorescent when compared to the fluorescence of the control population (HEK-WT).

Supporting Information 5: Chemokine secretion profile and ACKR scavenging of supernatant from CAFs isolated with alternative method

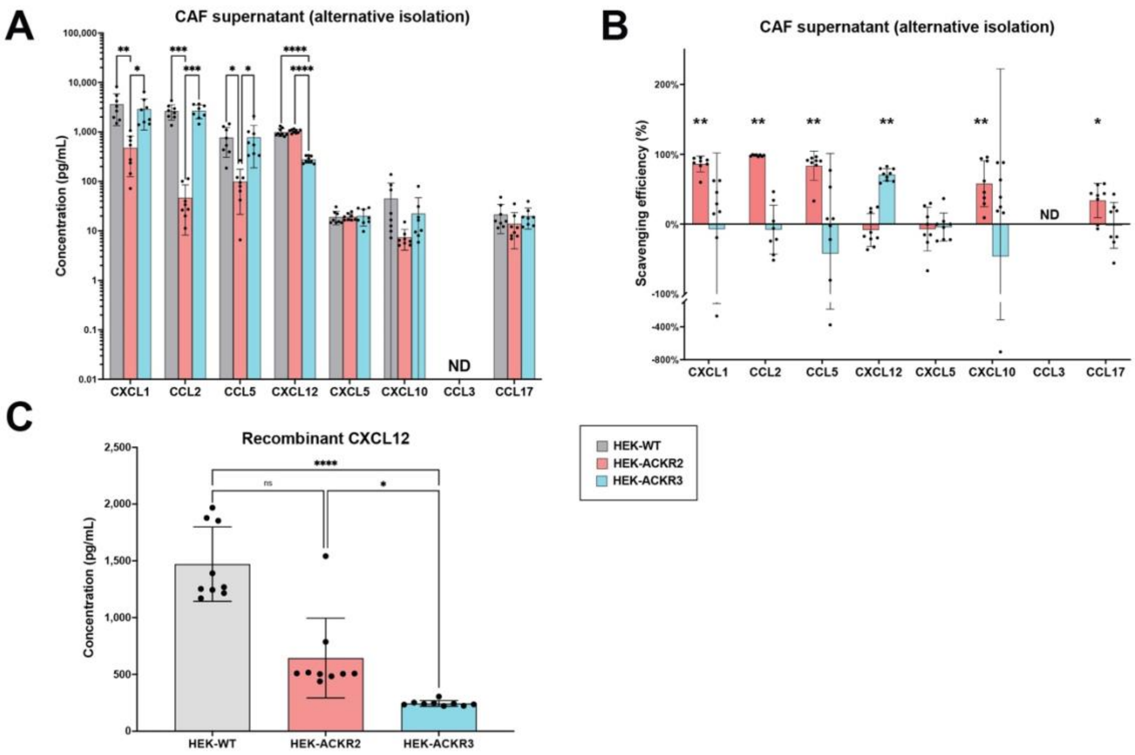

Supporting Information Figure 2: CBA of supernatants from CAFs isolated using an alternative method and absolute concentrations of CBA of recombinant CXCL12. (A) 3 independent biological replicates of supernatant were collected from confluent CAFs isolated using an alternative method (see Methods) after 72 h in 5 mL of DMEM media. Collected CAF supernatants were then incubated with 3 independent biological replicates of HEK cells as previously for 12 h, before chemokine concentrations were analysed by 13-plex proinflammatory and CXCL12 CBA panels. Chemokines that were below detectable limits are not shown. Data is shown as mean ± SD (error bars) collected from 2 independent biological replicates with measurements performed in triplicate and 1 independent biological replicate performed in duplicate for proinflammatory panel and 3 independent biological replicates performed in triplicate for CXCL12 panel. p-values by two-way ANOVA with Tukey's multiple comparisons test, ns not shown, * p>0.05, ** p<0.05, *** p <0.01, ****p <0.001 **(B)** Scavenging efficiency (%) was calculated for samples in (A). p-values by Wilcoxon matched pairs two-tailed test from a theoretical median of 0; ns not shown; ** p>0.01. **(C)** Absolute concentrations for the recombinant CXCL12 CBA shown in Figure 2C. p-values by Kruskal-Wallis test with Dunn's multiple comparisons; ns p>0.05; * p>0.05; ****p <0.001.

Supporting information 6: Details of chemotaxis migration assay

The µ-Slide chemotaxis coverslip was used to quantify CTL chemotaxis in a 3D collagen gel matrix in the presence of KPC CAF and ACKR2 expressing or untransduced HEK cells over 16hrs. Each coverslip has 3 chambers, with each chamber consisting of a central channel (1mm x 70µm) connected to two flanking side reservoirs. Each central chamber and side reservoirs have two opening ports that can be filled with plugs to equalise pressure within the chamber. CTLs are embedded in a collagen gel in the central chamber, with CAF and HEK cells seeded in one side reservoir to establish a chemokine gradient to the other side reservoir. Supporting Information Figure 3 details the principles of the µ-Slide chemotaxis coverslip and experimental set-up used.

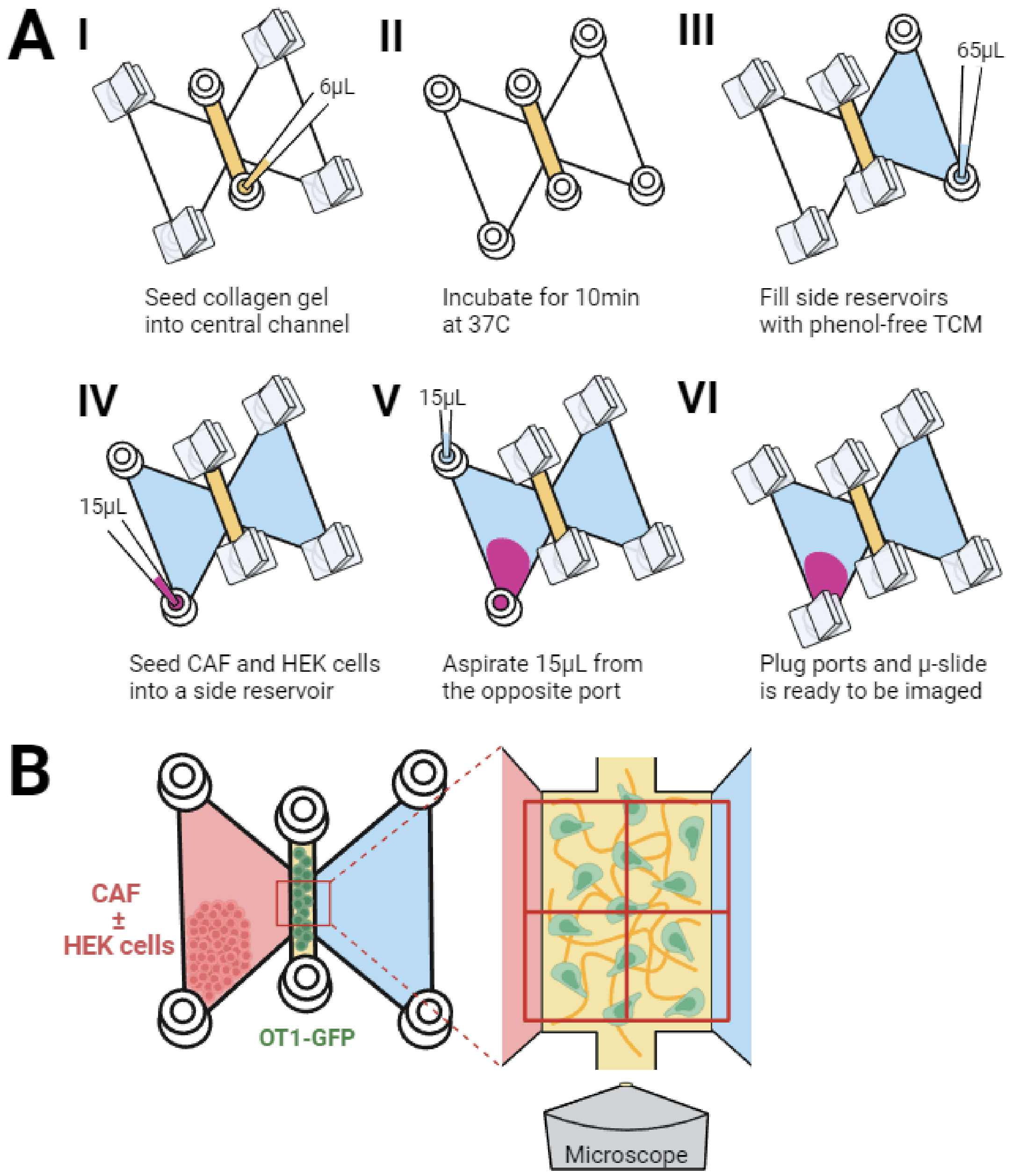

Supporting Information Figure 3. Principle and set up of the µ-Slide chemotaxis assay. (A) The experimental sequence is shown. (I) A low-density collagen gel mix containing 5×105 OT1-GFP cells

was prepared in 38µL of phenol-free TCM, 10µL of 1x PBS, 1.44µL of 1N NaOH and 50µL of rat tail collagen I (3.38mg/mL) (Corning). 6µL of low-density collagen gel is seeded into the central channel of a chamber. (II) 10min incubation at 37°C and 5% CO_2 to allow the collagen gel to set. (III) Side reservoirs are filled one at a time with 65µL of phenol-free TCM with the opposite reservoir ports plugged. (IV) 15µL of the 3.3×10^6 CAF and HEK cells mixed in a 1:1 ratio is pipetted into a side reservoir, (V) and 15µL is immediately aspirated from the opposing port, creating a temporary pressure differential that pulls the cells inside the reservoir. (VI) All ports are plugged, and µ-Slide chemotaxis coverslip are imaged using Leica SP8 DLS with a 10x objective lens set at 0.75x zoom, 600Hz scan speed and 512x512 resolution. A 488nm laser was used to excite OT1-eGFP cells and brightfield was used to image the bounds of the central chamber. **(B)** Schematic of an experimental condition chamber layout with OT1-eGFP cells seeded in collagen gel in the central chamber with KPC CAF cells and HEK cells in a side reservoir. Each chamber was imaged simultaneously using 4 overlapping tiles with a 15% overlap, shown as red squares in figure, with a ~60µm Z-depth and 3µm step size per chamber. Images were merged upon acquisition on the Lecia SP8 DLS and image analysis was conducted using Imaris software version 9.2.1. All FOVs were cropped to the same size (975x627 pixel size, 2957x1901µm) and spots were created corresponding to GFP fluorescence with an estimated diameter of 10µm and above a background threshold subtraction quality of 8

Supporting References

1. Maetzig T, Galla M, Baum C, Schambach A. Gammaretroviral vectors: biology, technology and application. Viruses. 2011;3(6):677-713.

2. von Stetten D, Noirclerc-Savoye M, Goedhart J, Gadella TWJ, Jr., Royant A. Structure of a fluorescent protein from Aequorea victoria bearing the obligate-monomer mutation A206K. Acta Crystallogr Sect F Struct Biol Cryst Commun. 2012;68(Pt 8):878-82.

3. Galeano Niño JL, Pageon SV, Tay SS, Colakoglu F, Kempe D, Hywood J, et al. Cytotoxic T cells swarm by homotypic chemokine signalling. eLife. 2020;9:e56554.

4. Gill SC, von Hippel PH. Calculation of protein extinction coefficients from amino acid sequence data. Anal Biochem. 1989;182(2):319-26.

5. Pace CN, Vajdos F, Fee L, Grimsley G, Gray T. How to measure and predict the molar absorption coefficient of a protein. Protein Sci. 1995;4(11):2411-23.

6. Overton WR. Modified histogram subtraction technique for analysis of flow cytometry data. Cytometry. 1988;9(6):619-26.
